# Nutritional Status of Children 24–60 Months Attending Early Child Development Centres in a Semi-Rural Community in South Africa

**DOI:** 10.3390/ijerph18010261

**Published:** 2020-12-31

**Authors:** Onwaba Makanjana, Ashika Naicker

**Affiliations:** Department of Consumer Sciences: Food and Nutrition, Durban University of Technology, 70 Steve Biko Road, Musgrave, Berea 4001, South Africa; ashikan@dut.ac.za

**Keywords:** nutritional status, Early Childhood Development, stunting

## Abstract

Despite the numerous efforts to improve the nutritional status of children, a high prevalence of malnutrition still exists in South Africa. This study aimed to determine the nutritional status of children attending Early Child Development centres in South Africa. In this baseline study, we randomly selected two Early Child Development centres comprising 116 children aged 24–60 months, separated into two cohorts, of 24–47 months and 48–60 months. Dietary intake was measured through the 24 hDR and analysed using Food Finder software. The food frequency questionnaire was used to calculate the food variety and food group diversity scores. Anthropometric measurements were taken and the WHO Anthro software was used to convert it to nutritional data indices. Blood samples were collected through dried blood spot cards in order to determine serum retinol and haemoglobin levels and they were assessed using WHO indicators. The findings showed that participants between 24 and 47 months had a high mean energy intake (4906.2 kJ and 4997.9 kJ for girls and boys, respectively). For the 48–60 months age group, energy intake was lower than the EER (5936.4 kJ and 5621.2 kJ; *p* = 0.038). There was low fruit and vegetable consumption (24–47 months; 63.8 g and 69.5 g (*p* = 0.037), 48–60 months; 68.3 g and 74.4 g (*p* = 0.038) and the top five foods consumed were carbohydrate rich foods for girls and boys, respectively. Stunting was noted in 7% and 20% (48–60 months) (*p* = 0.012) and overweight in 8% and 17% (24–47 months) and 17% and 13% (48–60 months) (*p* = 0.041) in girls and boys, respectively. Low serum retinol levels (<0.070 µmol/L) were found in 9.1% of boys (24–47 months), and 8% and 7.4% of girls and boys (48–60 months), respectively. Low haemoglobin levels (<11.0 g/dL) were found in 50.0% and 30.4% (24–47 months) and 8.6% and 39.3% (48–60 months) of girls and boys, respectively. Malnutrition, despite many national and provincial initiatives, still exists in Early Childhood Development centres in South Africa, calling for the application of contextualized nutrition interventions to suit resource-poor settings.

## 1. Introduction

The early childhood period is the most crucial developmental phase in life, and thus, the ultimate aim is for all children to be free of malnutrition in all forms. The six global targets set by the World Health Assembly in 2012 included a 40% reduction in the number of stunted children by 2025, no increase in childhood overweight and maintenance of the prevalence of wasting to less than 5% [1]. The global agenda to improve nutrition by 2030 aims to end all forms of malnutrition, reduce stunting by 50% and reduce childhood overweight and wasting numbers to less than 3% [2]. Globally in 2018, 821.6 million people were undernourished, of which 256.1 million were from Africa. Moreover, 9.2% of people worldwide experienced hunger and Africa accounted for the highest percentage (21.5%) of people experiencing severe food insecurity [3]. It has been well established that stunting is the devastating result of poor nutrition in-utero and early childhood. Even though progress has been made in various regions to reduce stunting, almost 149 million children below five years of age are stunted [3]. As the emerging face of childhood malnutrition moves towards overweight and obesity conditions, the prevalence of overweight in children under five has increased dramatically with 40.1 million identified as overweight [3]. While wasting in children is seen as the life-threatening result of sub-optimal nutrient intake and/or disease, in 2019, wasting was prevalent among 49.5 million children [3].

Various studies show that the diet of the South African population, particularly children, is generally low in fruit and vegetables, and mostly consists of starch-based foods [4,5,6]. Despite numerous efforts to improve the nutritional status of South African children, stunting is the most common nutritional disorder similar to global trends in undernutrition [7]. Additionally, the South African National Health and Nutrition Examination Survey (SANHANES-1) showed that 43.6% of children under five years had vitamin A deficiency and 10.7% had anaemia in 2013; however a marked reduction of the prevalence of anaemia was noted from 2005 onwards [8]. Good nutrition in the first 1000 days allows children to survive, grow, develop, learn, play, participate and contribute to society. Notable among gaps in the achievement in the nutritional status of children in South Africa lies the potential to introduce contextualised interventions through a syndemic approach involving the interacting nature of nutritional status and the social and environmental factors that promote their negative relationship [9].

Interventions to improve the health and nutritional status of children need to be implemented as early as possible, to reduce the effects of malnutrition on growth and development of young children. The Department of Social Development (DSD) identified Early Childhood Development (ECD) centres as an ideal platform for implementing strategies aimed at reducing poverty in children below five years of age in South Africa. ECD centres are facilities designed to provide early childhood development services and programmes that include good health, proper nutrition and early learning through a holistic approach in promoting a healthy environment that is conducive for learning and development [10]. ECDs in South Africa are non-profit organizations that are funded by the department of social development and the department of education [11]. The General Household Survey by Statistics South Africa indicated that over 2 million children receive some form of ECD services. Of this number, 800,654 children are currently accessing registered ECD programmes by the Department of Social Development and 626,574 of these children receive a subsidy of R17 per child per day. There are currently 14,205 registered partial care facilities that operate as ECD centres while many remain unregistered as they do not comply with the minimum norms and standards set out in the Children’s Act [12]. Given the strong subscription of ECDs in South Africa, ECDs are the perfect platform to continue support for the first 1000 days of life. The arguments in favour of promoting the development of children at a very young age are clear and compelling. Against this backdrop, the study aims to determine the nutritional status of 24–60 months old children attending ECD centres in South Africa for the purposes of planning robust and contextualised nutritional interventions suited to resource-poor settings.

## 2. Materials and Methods 

### 2.1. Study Design and Sampling

This study formed part of a randomised control trial, of which the baseline results for the entire group are presented in this paper. Children between the ages of 24 to 60 months attending two registered ECD centres were eligible for the study. Simple random sampling techniques were used to select two ECD centres from a list of ECD centres in the Valley of a Thousand Hills in Durban. The Valley of Thousand Hills is a semi-rural area located 40 km from Durban in KwaZulu-Natal (KZN), South Africa. The population size of the area where the study was conducted was 815 (33.70 per km^2^) [13]. The study sample consisted of 116 children between the ages of 24–60 months separated into two cohorts (24–47 and 48–60 months). 

### 2.2. Dietary Intake

Dietary intake data was measured using a validated 24 h dietary recall questionnaire (administered three times: two weekdays and one weekend day) and a Food Frequency Questionnaire (FFQ) [14]. Trained fieldworkers administered the questionnaires to the parent or caregiver of the child. Food samples were used to aid portion size identification. 

### 2.3. Anthropometric Measurements

Anthropometric measurements including height (up to the nearest 1 mm using the SECA stadiometer) and weight (up to the nearest gram using the SECA weighing scale, calibrated before weighing session) were taken using WHO standard indicators to assess wasting, stunting, underweight, overweight and obesity [15]. All measurements were recorded twice using standardised prescribed anthropometric measurement procedures [15]. 

### 2.4. Biochemical Measures 

Using the finger-prick method, blood samples (50µL) were collected, and dried blood spots (DBS) were prepared by trained health professionals to determine biochemical indicators of vitamin A and haemoglobin (Hb) levels of the participants. The participants washed their hands and rubbed them together to generate warmth to promote blood circulation. A pea-size amount of a topical anaesthetic cream was applied to the fingertip 20 min before pricking. The fingertip was thereafter pricked with a sterile retractable lancet and blood drops were allowed to fall freely inside the pre-printed circles on labelled DBS cards. The DBS cards were then placed in drying racks and left to dry at room temperature. All safety protocols for blood collection were observed [16]. After drying, the DBS cards were placed inside Ziploc plastic bags from the DBS collection kit, and a desiccant sachet was placed inside the cooler boxes and transported to the laboratory in a temperature-controlled environment. The samples were thereafter stored at −80 °C until analysis [16]. Biochemical indicators of vitamin A were assessed using High Performance Liquid Chromatography (HPLC) and vitamin A levels < 0.070 µmol/L were considered as vitamin A deficiency [17]. Haemoglobin levels were measured using a haemoglobin meter and compared to prescribed cut-offs (<11.0 g/dL) for iron deficiency for the age group [18].

### 2.5. Ethics Approval and Consent to Participate

Ethical approval was granted by the Institutional Research Ethics Committee at Durban University of Technology (IREC no 019/13). Gatekeeper permission to conduct the research was sought from relevant stakeholders. A letter of information in English and in isiZulu (local language) was shared and explained to all internal gatekeepers, parents and caregivers. Written consent was obtained from the principals of the ECD centres to conduct the study at their facilities and from parents or caregivers of the child. All biomedical waste was disposed of using biomedical waste management rules [19].

### 2.6. Data Management and Analysis

Data from the 24hDR was captured and analysed using Food Finder Software (version 3) to determine mean nutrient intake and the top ten foods consumed [20]. The nutrient intake was compared to the Recommended Dietary allowance (RDA) and AI for the age group 24–47 months and 48–60 months (15). The FFQ data were used to determine the food variety score (FVS) and food group diversity scores (FGDS). The mean fruit and vegetable intake were compared to the South Africa Peaediatric guidelines for children aged 1–7 years [21]. Anthropometric data were analysed using the WHO Anthro software version 3.2.2 [22]. Stunting was defined as a low length/height-for-age z-score (<−2 for moderate or <−3 for severe), wasting as a low weight-for-height z-score (<−2 for moderate or <−3 for severe), and underweight as low weight-for-age z-score (<−2 for moderate or <−3 for severe). The data were analysed for descriptive statistics and independent *t*-tests using the Statistical Package for the Social Sciences SPSS^®^ statistical software package Version 24.0 (IBM SPSS Inc., Chicago, IL, USA) for analysis. *p* values <0.05 were considered statistically significant. 

## 3. Results

### 3.1. Participant Profile

The sample size comprised of 116 children of which there were 58 children in 24–47 months category (girls *n* = 30, boys *n* = 28) and 58 children in the 48–60 months category (girls *n* = 28, boys *n* = 30).

### 3.2. Nutrient Intake

Table 1 illustrates that the mean energy intake for girls (4906.2 kJ) and boys (4997.9 kJ) between 24–47 months was above the estimated energy requirement (EER). In the 48–60 months age group, the mean energy intake was 5936.4 kJ for girls and 5621.2 kJ for boys (*p* = 0.038). The mean intake of protein was 33.1 g for girls and 36.7 g for boys between 24–47 months (*p* < 0.001) and was 41.3 g and 39.2 g for girls and boys, respectively, for the 48–60 months age group (*p* < 0.001). The mean intake of carbohydrates was significantly higher than the recommended amount for both age categories; 167.5 g and 176.9 g for girls and boys respectively between 24 and 47 months (*p* = 0.011) and 201.1 g for and 193.5 g for girls and boys between 48 and 60 months. The contribution of fat to total energy was 26.7% and 25.8% in girls and boys between 24 and 47 months respectively. In the 48–60 months age group, fat contributed 26.7% to total energy for girls and 25.2% for boys. In contrast, the mean dietary fibre intake was lower than the recommended amount; 10.7 g for girls and 13.4 g for boys between 48 and 60 months, 13.6 g for girls and 14.4 g for boys aged 40–60 months. Ninety-seven percent of girls and 86% of boys between 24 and 47 months did not meet the daily recommended amount for calcium, with the mean intake being lower in girls (248.3 mg) than boys (307.1 mg) (*p* < 0.001). The mean intake of calcium for participants between 48–60 months was also lower than the recommended intake, (346.9 mg for girls and 241.2 mg for boys (*p* = 0.056).

The results also show that the mean intake of vitamin A was higher than the recommended RDA for all participants between 24–47 months (456.2 µg and 551.3 µg for girls and boys respectively) (*p* < 0.001). A similar trend was observed in girls (608.7 µg) and boys (506.3 µg) between 48 and 60 months (*p* = 0.019). All boys between 24 and 47 months met the RDA for folate per day; however, 7% of girls in that age group consumed less than the recommended amount. In the 48–60 months age group, results show that even though the mean intake of folate was higher for girls (251.8 µg) compared to boys (273.0 µg), 11% of girls consumed less than the RDA (*p* < 0.001). This trend was also noted for vitamin B12 for participants aged 48–60 months where the mean intake was 2.8 µg for girls and 2.4 µg for boys (*p* = 0.031); however, 7% of girls consumed less than the RDA.

The results presented in Table 2 indicate that for the 24–47 months age group, the mean fruit and vegetable intake for girls was 63.8 g and 69.5 g for boys (*p* = 0.037). In the 48–60 months age group, the mean was 68.3 g for girls and 74.4 g for boys (*p* = 0.038). Results also showed that the mean FVS was 34 for participants between 24 to 47 months and 38 for participants between 48 to 60 months, indicating medium variety. The mean FGDS was 8.2 for the 24 to 47 month age group and 8.5 for 48 to 60 months age group.

Table 3 shows that maize meal was the most consumed item among girls and boys between 24–47 months (2491.1 g and 2577.6 g respectively), followed by diluted squash cold drink (1695.8 mL for girls and 1408.1 mL for boys) and rice at third place (1468.3 g and 860.8 g). A donated instant vanilla porridge was placed fourth for both groups (942.3 g for girls and 865.0 g for boys) and bread at number five (691.6 g and 1654.1 g for girls and boys respectively). The top three foods consumed by girls and boys between 48 and 60 months were maize meal (2151.3 g for girls and 3086.1 g for boys), rice (1576.0 g for girls and 1770.9 g for boys) and diluted squash (1547.0 mL and 1091.6 mL for girls and boys respectively).

### 3.3. Anthropometric Status

**Figure 1:**
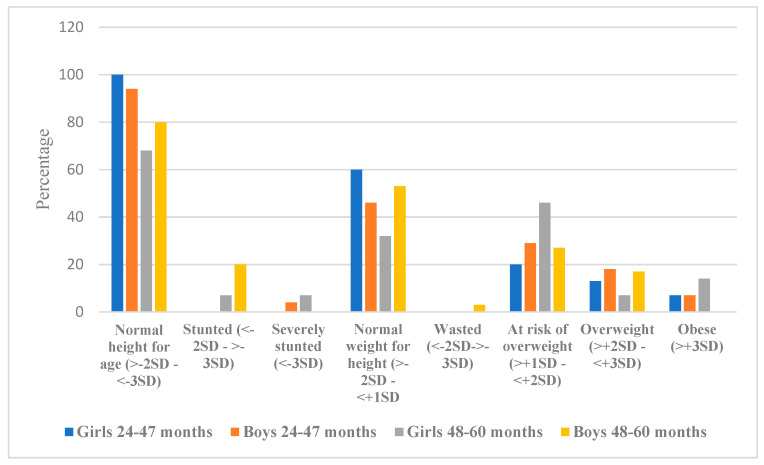
Girls’ and boys’ anthropometric results (*n* = 116).

Figure 1 illustrates that the majority of the participants had a normal height for age in both groups. Stunting was noted in 7% of girls and 20% of boys between 48 and 60 months (*p* = 0.012), and 7% of girls were severely stunted, as per WHO standards (19). The percentage of girls with a normal weight for height was lower in the 48–60 months age group (32%) compared to girls between 24 and 47 months (60%). Among boys, 46% in the 24 to 47 months and 53% in the 48 to 60 months age group, a normal weight for height was recorded. Wasting was observed in 3% of boys between 48 and 60 months. The risk of being overweight was higher in girls between 48 and 60 months (46%) compared to 27% of boys in the same age group (*p* = 0.026). In contrast, more boys (29%) were at risk of being overweight compared to girls (20%) in the 24 to 47 months age group. Eighteen percent of boys between 24 and 47 months were overweight, closely followed by boys between 48 and 60 months at 17% and girls between 24 and 47 months at 13%. Results showed a statistically significant difference between overweight girls and boys between the ages of 48 to 60 months (*p* = 0.041). Seven percent of girls and 7% of boys between 24 to 47 months were obese. In the 48 to 60 months age group, 14% of girls between were obese, and none of boys were obese in that age group (*p* = 0.004).

### 3.4. Vitamin A and Iron Status

For the biochemical measurements, the sample size decreased to *n* = 90 due to 15 participants being absent on blood collection days and the inability to detect serum retinol in *n* = 11 of the samples (refer to Table 4). Low serum retinol levels (<0.070 µmol/L) were found in 9.1% of boys between 24 and 47 months, and none of the girls had low serum retinol levels in this age group. Eight-point three percent girls and 7.2% of boys between 48 to 60 months had low serum retinol levels. In the 24 to 47 months age group, 50.0% of girls and 30.4% of boys had low Hb levels (<11.0 g/dL) whereas in the 48 to 60 months age group, low Hb levels were found in 28.6% of girls and 39.3% of boys.

## 4. Discussion

In general, the study findings showed that participants between 24 and 47 months had a higher mean energy intake whereas those in the 48 to 60 months age group had a low mean energy intake compared to EER for that age group. There was low fruit and vegetable consumption, high prevalence of stunting, overweight and iron deficiency. The importance of nutrition in early childhood development and the effects of inadequate nutrition beyond childhood has been well established [24]. Results in this study show that the fibre and calcium intake were lower than the recommended amount in all the age groups, similar to findings in a study conducted in Durban in South Africa [6]. The contribution of fat to total energy intake was less that the recommended 30–40% for children between 24 and 47 months. However, for children between 48 and 60 months, it was within the recommended Acceptable Macronutrient Distribution Range (AMDR) of 25–33%. Furthermore, even though the mean dietary intake was higher than the RDA for vitamin A in all groups, low serum retinol levels were found in some boys between 24 and 47 months and in both girls and boys between 48 and 60 months. Similarly, low Hb levels were found in all age groups, despite dietary intake indicating adequate iron intake. This highlights that dietary intake results from a 24 h dietary recall should be interpreted with caution as some participants may underreport or incorrectly estimate their food intake despite using food samples during data collection. Moreover, there was generally a high protein intake in all groups, and none of the participants consumed less than the recommended intake. Given that the food intake results are similar to findings reported in the National Food Consumption Survey conducted in 1999 and a more recent study at an ECD centre in a similar context [6,7], and despite national interventions, various reasons can be attributed to the current results. Nutritional status is influenced by several environmental factors and, as such, in countries like South Africa where the prevalence of HIV infection is high, HIV infection has both a direct impact on the nutritional status of women and children who are infected and an indirect effect through changes in household food security and inappropriate choices of infant-feeding practices to prevent mother-to-child transmission of HIV [25].

It has been well established that adequate fruit and vegetable consumption is crucial for child health. Globally and nationally, most children do not meet the guidelines for adequate fruit and vegetable intake. The results from this study are no different; the mean fruit and vegetable intake for all participants between 24 and 47 months and 48 to 60 months was less than the recommended 320 to 480 g per day and 400 to 480 g per day respectively. These findings of low fruit and vegetable intake compare with the SANHANES-1 study. Cost is the major constraint prohibiting daily consumption of fruits and vegetables [26]. Given that 65% of young children in South Africa live below the poverty line, affordability is related to the low fruit and vegetable intake [27]. Although initiatives such as food gardens have been amplified and promoted as a nutrition intervention strategy, its impact is seldom measured. Moreover, the dislike of fruit and vegetables among children and the habit of eating fruits and vegetables at an early age is another possible reason. According to Raggio and Gambaro, (2018) the sensory characteristics of vegetables and the habits of consumption in the family environment play an important role in acceptance or rejection of vegetables by children [28]. 

Anthropometric results show that over and undernutrition coexists within the study population, which reflects similar trends in developing countries [29]. The incidence of stunting was noted in girls and boys between 48 and 60 months, with severe stunting being more pronounced among girls. The high prevalence of stunting in this study population is similar to other studies in South Africa [30,31]. While wasting was only observed in boys aged 48 to 60 months, the risk of overweight was high in all age groups, especially in girls between 48 and 60 months. The anthropometric results indicating the prevalence of overweight in this study link with the dietary intake results, as the group with higher mean energy intake (24–47 months) had a higher percentage of participants who were overweight. The more recent South African Demographic Health Survey in 2016 found a decline in wasting and underweight, yet stunting remained high, affecting 27% of children under five [32]. Stunting is an indicator of chronic malnutrition compromising children’s cognitive development, education and employment prospects, and increases their risk of overweight and obesity [25]. While South Africa has experienced a rapid nutrition transition characterised by an increase in the prevalence of obesity and non-communicable diseases, the South African pattern of transition differs in that stunting persists [33]. To effectively direct public health initiatives, an understanding of the long-term dynamics of stunting in the South African context is required in conjunction with the interplay of the obesogenic food environment.

In children, vitamin A is essential to support rapid growth and helps to combat infections (WHO). The findings in this study show that some of participants had low serum retinol levels. When comparing vitamin A dietary intake and serum retinol concentration similar results were noted. For girls 24–47 months, both methods recorded the same results (100% of the DRI for vitamin A and 100% serum retinol level > 0.070 µmol/L). For all other age groups a close difference between dietary vitamin A and serum retinol level was reported; boys 24–47 (9.1% and 14% lower, girls 48–60 (8.3% and 11%), boys 48–60 (7.2% and 22%) were lower than the DRI for vitamin A and serum retinol level < 0.070 µmol/L. In the SAHANES study at the national level, the prevalence of vitamin A deficiency was 43.6%. South Africa introduced routine periodic high-dose vitamin A supplementation in 2002 to reduce childhood mortality, however, there is no evidence from the past two decades, with changing disease profiles, increased use of vaccines and reduced morbidity from diarrhoea and pneumonia, that a high-dose programme is nearly as effective today as it was in some countries 20 to 30 years ago [34]. Moreover, it has been found that there may also be pockets where, due to unique eating patterns, vitamin A deficiency may not be present at all [35]. 

Iron deficiency can have a serious impact on children’s health and later development through alteration of the immune status, adverse effects on morbidity and delayed behavioural and mental development [36]. In this study, the prevalence of anaemia was high contrary to the findings of the SAHANES study where the overall prevalence of anaemia was 10.7%, mild anaemia 8.6% and moderate anaemia 2.1% [8]. It was also found that children in the 24–47 months age group had lower Hb levels. Likewise, the prevalence of anaemia was highest in children in the 24–35 months (15.2%) age group and decreased to 3.0% in the 48–59 months age group in the SAHANES study [8]. The mean iron intake of participants seemed adequate however, low Hb levels were noted in all groups. A possible reason for the low Hb levels in this study population could be alluded to inaccurate reporting of food intake or dietary bioavailability. Co-morbid anaemia and stunting among young children are highly prevalent in low- and middle-income countries [9]. Hence a syndemic framework approach is encouraged integrating the co-occurrence of health problems with social and environmental factors.

## 5. Limitations

This study is not without limitations. The results of this study represent a semi-rural geographical area which may not represent a typical rural setting. Using a finger prick to draw blood samples posed limitations as serum retinol could not be detected in some samples. Researchers could not draw blood intravenously as that would be invasive given the age of participants. Other limitations included a small sample size and potential recall bias.

## 6. Conclusions

Despite the numerous efforts to improve the nutritional status of children, a high prevalence of malnutrition still exists in South Africa. Malnutrition, presented through micronutrient deficiencies, stunting, overweight and obesity, co-exist in this study population. The nutritional status of children’s diet, attending ECD centres, is sub-optimal and is characterised by inadequacies for optimum growth with a marked increase in the prevalence of stunting, overweight and low haemoglobin levels. 

## 7. Recommendations

Early interventions to address both under and overnutrition are required. Menus should be reviewed to include more fruit and vegetables and unrefined carbohydrates. Sustainable food-based interventions should be explored with the intent to support good health, proper nutrition and early learning at ECD centres towards the optimum development of human capital. The co-morbidity of anaemia and stunting in early childhood resource-constrained settings should be managed using a syndemic approach. Sustainable interventions should be explored with the intent to support good health, proper nutrition and early learning at ECD centres, towards the optimum development of human capital.

## Figures and Tables

**Table 1:** Mean dietary intake for girls and boys (*n* = 116) 24–60 months as measured by the 24 h food recall.

Girls and Boys 24–47 Months (*n* = 58)						Girls and Boys 48–60 Months (*n* = 58)				
Nutrient	Girls Mean Nutrient Intake *n* = 30 (SD)	% Girls < 100% of DRI	Boys Mean Nutrient Intake *n* = 28 (SD)	% Boys < 100% of DRI	*p* Value	Girls Mean Nutrient Intake *n* = 28 (SD)	% Girls < 100% of DRI	Boys Mean Nutrient Intake *n* = 30 (SD)	% Boys < 100% of DRI	*p* Value
Energy kJ *	4906.2 (292.6)	40	4997.9 (734.7)	17.8	0.541	5936.4 (701.4)	82	5621.2 (339.8)	96.6	**0.038**
Protein (g) **	33.1 (2.9)	0	36.7 (4.2)	0	**0.001**	41.3(0.4)	0	39.2 (0.9)	0	**0.000**
CHO (g) **	167.5(5.1)	0	176.6(17.1)	0	**0.011**	201.1 (36.1)	0	193.5 (15.7)	0	0.316
Total fat (g)	35.4 (18.4)	-	34.9 (9.3)	-	0.842	42.8 (30.4)	-	38.3 (9.4)	-	0.342
Total dietary fibre (g) ***	10.7 (1.3)	100	13.4(1.4)	82	0.307	13.6 (1.9)	100	14.4(1.7)	100	0.118
Calcium (mg) ***	248.3 (20.6)	97	307.1 (7.6)	86	**0.000**	346.9 (40.9)	100	241.2 (288.7)	97	0.056
Iron (mg) **	8.4 (1.0)	0	9.9(0.9)	0	**0.000**	10.4 (1.3)	0	10.6(1.2)	0	0.641
Zinc (mg) **	6.15 (0.7)	0	7.7 (0.0)	0	**0.000**	8.1 (0.9)	0	7.9 (1.2)	0	0.538
Vitamin A (µg) **	456.2 (59.6)	0	551.3 (85.3)	14	**0.000**	608.7 (134.4)	11	506.3 (186.0)	23	**0.019**
Riboflavin (mg)	1.6 (0.0)	0	1.5 (0.0)	0	**0.000**	2.2 (1.0)	0	2.1 (0.4)	0	0.477
Vitamin B6 (mg) **	1.8 (0.1)	0	2.3 (0.1)	0	**0.000**	2.4 (0.6)	0	2.3 (0.7)	0	0.900
Folate (µg)	207.1 (32.4)	7	256.0 (20.2)	0	**0.000**	251.8 (25.2)	11	273.0 (10.9)	0	**0.000**
Vitamin B12 (µg) **	2.45(0.6)	0	2.6 (0.5)	0	0.164	2.8 (0.7)	7	2.4 (0.4)	0	**0.031**

^1^ DRI for 24–47 months [23]; DRI for 48–60 months: [23], SD: standard deviation; * EER, ** RDA: Recommended dietary allowance; *** AI: adequate intake; *p* values given in bold font indicate that the mean nutrient intake is significantly different from the RDA/AI.

**Table 2:** Mean fruit and vegetable intake for all participants (*n* = 116).

	Girls 24–47 Months (*n* = 30)		Boys 24–47 Months (*n* = 28)		*p* Value	Girls 48–60 Months (*n* = 28)		Boys 48–60 Months (*n* = 30)		*p* Value
	Mean Intake (g) (SD)	Per Capita	Mean Intake (g) (SD)	Per Capita		Mean Intake (g) (SD)	Per Capita	Mean Intake (g) (SD)	Per Capita	
**Fruit and vegetables**	63.8 (13.2)	74.0	69.5 (5.4)	74.5	**0.037**	68.3 (8.4)	98.8	74.4 (13.0)	94.4	**0.038**

**Table 3:** Top 10 foods consumed by all girls and boys (*n* = 116) between 24 and 60 months as measured by the 24-h food recall.

Girls 24–47 Months (*n* = 30)			Boys 24–47 Months (*n* = 28)			Girls 48–60 Months (*n* = 28)			Boys 48–60 Months (*n* = 30)		
ITEM	Mean Intake (g) (SD)	Capita Intake (g)	ITEM	Mean Intake (g) (SD)	Capita Intake (g)	ITEM	Mean Intake (g) (SD)	Capita Intake (g)	ITEM	Mean Intake (g) (SD)	Capita Intake (g)
Maize meal	2491.1 (2114.0)	83	Maize meal	2577.6 (2414.0)	92	Maize meal	2151.3 (294.6)	77	Maize meal	3086.1 (2621.7)	103
Diluted squash cold drink	1695.8 (866.2)	56	Diluted squash cold drink	1408.1 (1019.4)	50	Rice	1576.0 (731.6)	56	Rice	1770.9 (1419.2)	59
Rice	1468.3 (1393.9)	48	Rice	860.8 (1296.6)	31	Diluted squash cold drink	1547 (−27.8)	55	Diluted squash cold drink	1091.6 (408.9)	36
Instant porridge	942.3 (−548.2)	31	Instant porridge	865 (−229.5)	31	Instant porridge	1454.6 (803.2)	52	Milk	1455.8 (365.3)	49
Bread	691.6 (549.1)	23	Bread	1654.1 (469.0)	59	Bread	765 (−139)	27	Instant porridge	1027.3 (646.7)	34
Tea	654.1 (218.0)	22	Milk	914.3 (−984)	33	Milk	725.0 (370.0)	26	Bread	617.5 (124.9)	21
Milk	538.83 (526.3)	18	Tea	484.1 (−684.7)	17	Apple	457.0 (41.9)	16	Apple	283.3 (10.6)	9
Pilchard curry	384.5 (328.8)	13	Apple	430 (−438.4)	15	Chicken curry	275.0 (181.4)	10	Phuthu and maas	778.3 (601.5)	26
Sausage curry	375.0 (275.7)	13	Pilchard curry	243 (−343.6)	9	Savoury snack	265.1 (121.3)	9	Tea	425.3 (400.6)	14
Apple	360.0 (325.2)	12	Sausage curry	225 (−318.1)	8	Banana	248.1 (350.9)	9	Potato stew	231.1 (77.0)	8

**Table 4:** Serum retinol (*n* = 90) and Hb (*n* = 101) levels.

		Girls 24–47 Months (*n* = 17) for Serum Retinol, (*n* = 22) for Hb (%)	Boys 24–47 Months (*n* = 22) for Serum Retinol, (*n* = 23) for Hb (%)	Girls 48–60 Months (*n* = 24) for Serum Retinol, (*n* = 28) for Hb (%)	Boys 48–60 Months (*n* = 27) for Serum Retinol, (*n* = 28) for Hb (%)
Serum retinol levels	>0.070 µmol/L	100	90.9	91.7	92.6
	<0.070 µmol/L	0	9.1	8.3	7.2
Hb levels	>11.0 g/dL	50.0	69.6	71.4	60.7
	<11.0 g/dL	50.0	30.4	28.6	39.3

## Data Availability

Data available on request due to restrictions: ethical. The data presented in this study are available on request from the corresponding author. The data set are not publicly available due to ethical reasons and institutional data privacy rules.
